# Bis(ethyl­enedi­ammonium) μ-ethyl­enedi­aminetetra­acetato-1κ^3^*O*,*N*,*O*′:2κ^3^*O*′′,*N*′,*O*′′′-bis­[tri­oxidomolybdate(VI)] tetra­hydrate

**DOI:** 10.1107/S2414314624006679

**Published:** 2024-07-12

**Authors:** Lamine Yaffa, Dame Seye, Antoine Blaise Kama, Assane Toure, Cheikh Abdoul Khadir Diop

**Affiliations:** a Laboratoire de Chimie Minérale et Analytique (LACHIMIA), Département de Chimie, Faculté des Sciences et Techniques, Université Cheikh Anta Diop, Dakar, Senegal; b Department of Physics-Chemistry UFR Science and Technology Iba Der THIAM University of THIES Senegal; c Université Alioune Diop de Bambey UFR Sciences Appliquées et Technologies de l'Information et de la Communication (SATIC) Équipe Chimie des Matériaux Inorganiques et Organiques (ECMIO) Senegal; Benemérita Universidad Autónoma de Puebla, México

**Keywords:** crystal structure, edta complex, molybdenum oxide, ethyl­enedi­amine­tetra­acetic acid

## Abstract

The title compound, (C_2_H_10_N_2_)_2_[(C_10_H_12_N_2_O_8_)(MoO_3_)_2_]·4H_2_O, which crystallizes in the monoclinic *C*2/*c* space group, was obtained by mixing molybdenum oxide, ethyl­enedi­amine and ethyl­enedi­amine­tetra­acetic acid (H_4_edta) in a 2:4:1 ratio. The complex anion contains two MoO_3_ units bridged by an edta^4−^ anion. The midpoint of the central C—C bond of the edta^4−^ anion is located on a crystallographic inversion centre. The independent Mo atom is tridentately coordin­ated by a nitro­gen atom and two carboxyl­ate groups of the edta^4−^ ligand, together with the three oxo ligands, producing a distorted octa­hedral coordination environment. In the three-dimensional supra­molecular crystal structure, the dinuclear anions, the organo­ammonium counter-ions and the solvent water mol­ecules are linked by N—H⋯O_w_, N—H⋯O_edta_ and O—H⋯O hydrogen bonds.

## Abstract 2

The title compound is a binuclear complex of molybdenum with a ethyl­enedi­amine­tretra­acetate ligand bridging two MoO_3_ units.

## Graphical abstract

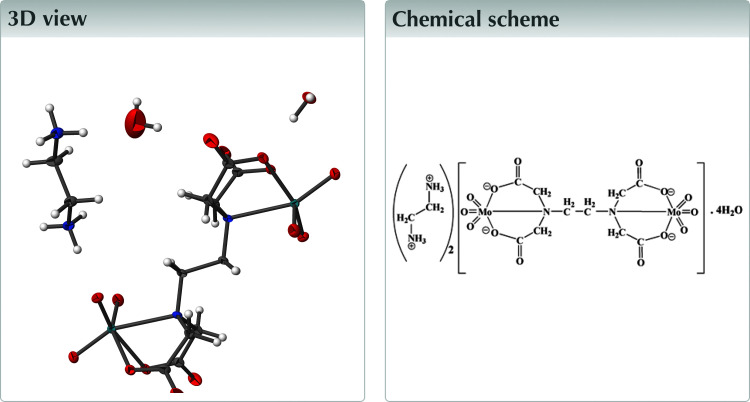

## Structure description

The advancement of materials science has meant that many well-established materials, such as metals, ceramics or plastics, cannot meet the demand for new applications (photovoltaic cells, field-effect transistors, *etc*.). This desire to design new functional materials demands enormous research effort. In order to overcome this challenge, scientists quickly understood that mixtures of materials could have properties superior to those of their pure counterparts, and thus meet this demand. Hybrid framework materials research is one of the fastest growing research fields.

Their unique hybrid nature enables the combination of properties from both inorganic and organic materials (Cheetham & Rao, 2007 ▸). As organic ligands, polycarboxyl­ates are multidentate chelating agents, widespread in nature and industry, due to their ability to coordinate to various transition metals in different ratios. In this field, the study of molybdenum polycarboxyl­ate complexes has led to thorough investigation over the past three decades. Some well-characterized mono-, bi- and polynuclear molybdenum and tungsten complexes have been reported, for example [(H_2_TEMED)Mo_2_O_6_(H_2_edta)]·H_2_O (TMED = tetra­methyl­ethylenedi­amine; Kumar *et al.*, 2012 ▸), Mo_2_(O_2_CCH_2_OH)_4_, *M*_2_[MoO_3_(C_2_O_4_)] (*M* = Na, K, Rb, Cs) and Na_2_[*M*O_2_(C_6_H_6_O_7_)_2_]·3H_2_O (*M* = Mo, W; Cotton *et al.*, 2002 ▸; Cindrić *et al.*, 2000 ▸; Zhou *et al.*, 1999 ▸), Na_2_K_2_[Mo_2_O_6_(edta)]·10H_2_O and Na_4_[W_2_O_6_(edta)]·8H_2_O (Lin *et al.*, 2006 ▸). In our study, the reaction of H_4_edta (ethyl­enedi­amine­tetra­acetic acid) with molybdenum oxide has been investigated, and a new binuclear 2:1 Mo–edta complex, (C_2_H_10_N_2_)_2_[(C_10_H_12_N_2_O_8_)(MoO_3_)_2_]·4H_2_O, including edta^4−^ as ligand has been isolated and structurally characterized.

**Figure 1:**
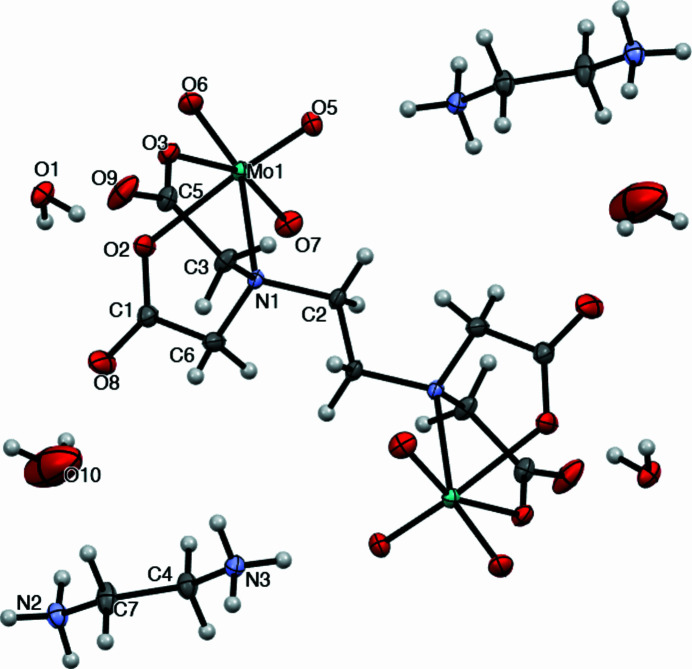
Mol­ecular structure of the title compound with displacement ellipsoids drawn at the 50% probability level. Unlabelled atoms are generated by inversion symmetry.

The single-crystal structure shows that the 2:1 Mo–edta complex anion of the title compound is discrete (Fig. 1 ▸). All of the carb­oxy­lic groups of H_4_edta are deprotonated, coordin­ating the molybdenum oxide groups by nitro­gen and two oxygen atoms. The edta^4−^ ligand itself is a bridge between the two MoO_3_ units, and the midpoint of the central C—C bond is situated on an inversion centre. In the 2:1 Mo–edta complex, the edta^4−^ ligand thus chelates a pair of Mo^VI^ centres, in a tridentate fashion, giving a *trans* configuration to the complex. Each Mo^VI^ ion is chelated by the edta^4−^ ligand, simultaneously forming two glycinato rings occupying contiguous vertices that define one face of the coordination polyhedron. The other three vertices of the opposite face are occupied by three terminal oxo atoms of the MoO_3_ unit, completing the octa­hedral geometry. In the complex, the Mo—O bond lengths are in the range 1.7195 (16) to 1.7686 (15) Å for Mo=O_t_ groups (O_t_ are terminal oxygen atoms: O5, O6 and O7). The resulting bond angles O_t_—Mo—O_t_ are 107.27 (7), 103.83 (7) and 106.75 (7)°, considerably larger than the expected value of 90° for a regular octa­hedron, confirming the distortion from octa­hedral geometry.

**Figure 2:**
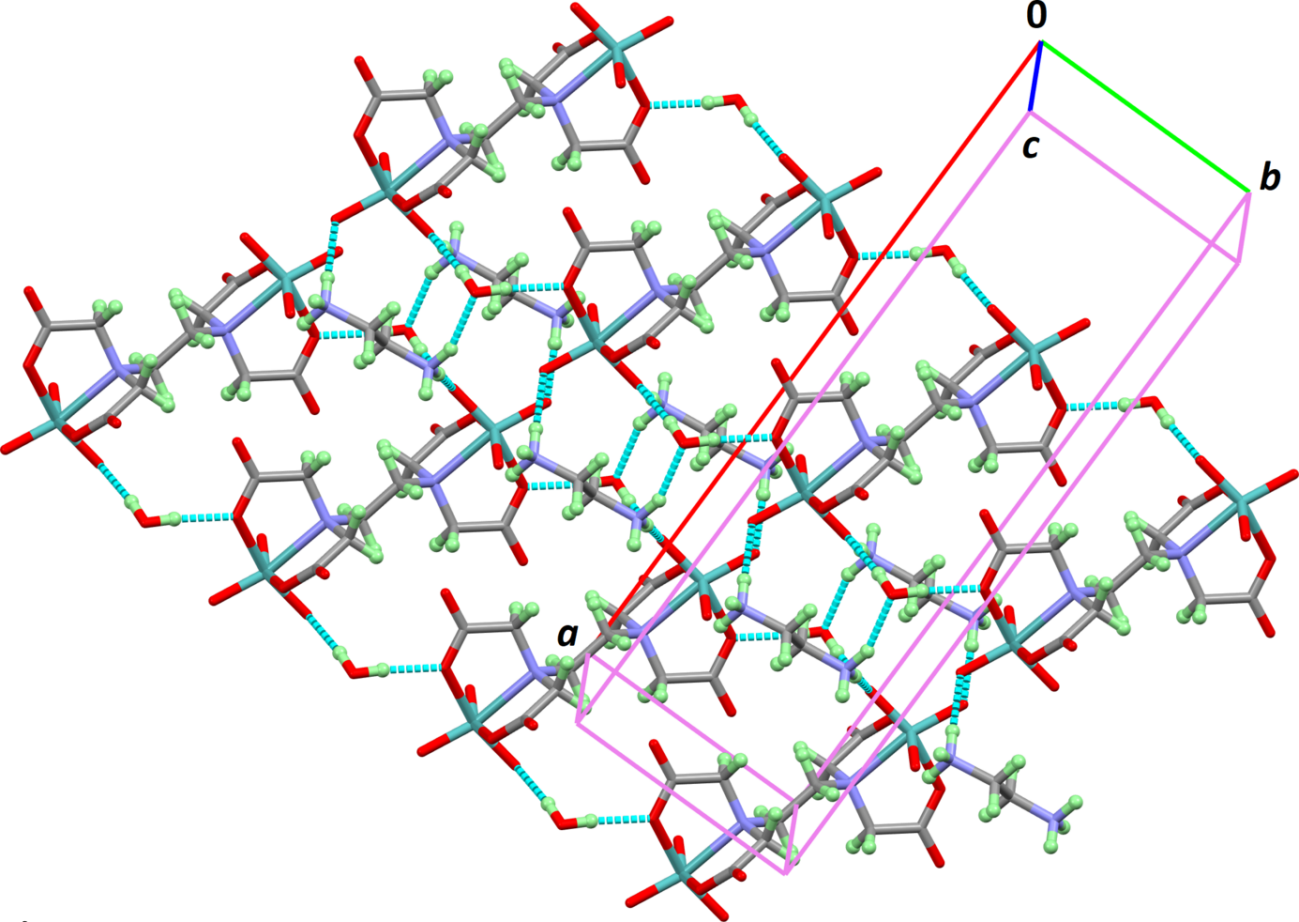
Supra­molecular arrangement of the title compound with hydrogen bonds shown as dotted lines.

The crystal packing can be rationalized in terms of non-bonding inter­actions between the three tectons: the Mo–edta complex anion, two (C_2_H_10_N_2_)^+^ cations and four lattice water mol­ecules. These units are linked through hydrogen bonds of the type N—H⋯O_water_, N—H⋯O_edta_ and O—H⋯O (Table 1 ▸). This inter­connection leads to the supra­molecular structure, as shown in Fig. 2 ▸.

## Synthesis and crystallization

Solid molybdenum oxide (4 mmol) and ethyl­enedi­amine (4 mmol) were mixed in 30 ml of distilled water. To this mixture were slowly added 2 mmol of ethyl­enediammine­tetra­acetic acid (H_4_edta) under vigorous stirring. The solution was then stirred for 2 h at room temperature. The colourless solution thus obtained was left at room temperature for slow evaporation of water. After a few days, colourless crystals (yield 13.6% based on Mo) were obtained.

## Refinement

Crystal data, data collection and structure refinement details are summarized in Table 2 ▸.

## Supplementary Material

Crystal structure: contains datablock(s) I. DOI: 10.1107/S2414314624006679/bh4085sup1.cif

Structure factors: contains datablock(s) I. DOI: 10.1107/S2414314624006679/bh4085Isup2.hkl

CCDC reference: 2368916

Additional supporting information:  crystallographic information; 3D view; checkCIF report

## Figures and Tables

**Table 1:** Hydrogen-bond geometry (Å, °)

*D*—H⋯*A*	*D*—H	H⋯*A*	*D*⋯*A*	*D*—H⋯*A*
N2—H2*C*⋯O1^i^	0.91	1.93	2.795 (2)	159
N2—H2*D*⋯O10	0.91	2.00	2.715 (4)	134
N2—H2*D*⋯O7^ii^	0.91	2.21	2.786 (2)	121
N2—H2*E*⋯O6^iii^	0.91	1.84	2.748 (2)	172
N3—H3*C*⋯O9^i^	0.91	1.88	2.785 (3)	170
N3—H3*D*⋯O1^iv^	0.91	1.98	2.838 (2)	156
N3—H3*E*⋯O5^v^	0.91	1.84	2.753 (2)	177
O1—H1*A*⋯O5^vi^	0.87	1.83	2.694 (2)	173
O1—H1*B*⋯O2	0.87	1.83	2.694 (2)	173
O10—H10*A*⋯O8	0.87	1.86	2.692 (4)	160
O10—H10*B*⋯O8^i^	0.87	2.13	2.978 (4)	166

Symmetry codes: (i) 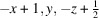; (ii) 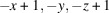; (iii) 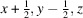; (iv) 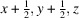; (v) 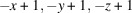; (vi) 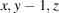.

**Table 2:** Experimental details

Crystal data	
Chemical formula	(C_2_H_10_N_2_)_2_[(C_10_H_12_N_2_O_8_)(MoO_3_)_2_]·4H_2_O
*M*_r_	772.40
Crystal system, space group	Monoclinic, *C*2/*c*
Temperature (K)	150
*a*, *b*, *c* (Å)	22.5897 (14), 7.5100 (4), 16.3743 (10)
β (°)	94.716 (2)
*V* (Å^3^)	2768.5 (3)
*Z*	4
Radiation type	Mo *K*α
μ (mm^−1^)	1.00
Crystal size (mm)	0.17 × 0.17 × 0.13

Data collection	
Diffractometer	Bruker APEXII CCD
Absorption correction	Multi-scan (*SADABS*; Krause *et al.*, 2015 ▸)
*T*_min_, *T*_max_	0.691, 0.746
No. of measured, independent and observed [*I* > 2σ(*I*)] reflections	24031, 3192, 2946
*R*_int_	0.032
(sin θ/λ)_max_ (Å^−1^)	0.650

Refinement	
*R*[*F*^2^ > 2σ(*F*^2^)], *wR*(*F*^2^), *S*	0.022, 0.060, 1.06
No. of reflections	3192
No. of parameters	189
H-atom treatment	H-atom parameters constrained
Δρ_max_, Δρ_min_ (e Å^−3^)	0.67, −1.06

Computer programs: *APEX2* and *SAINT* (Bruker, 2016 ▸), *SHELXT2018/2* (Sheldrick, 2015*a* ▸), *SHELXL2018/32* (Sheldrick, 2015*b* ▸), *Mercury* (Macrae *et al.*, 2020 ▸) and *publCIF* (Westrip, 2010 ▸).

## full crystallographic data

### Bis(ethylenediammonium) µ-ethylenediaminetetraacetato-1κ3O,N,O':2κ3O'',N',O'''-bis[trioxidomolybdate(VI)] tetrahydrate Crystal data

**Table:**

(C_2_H_10_N_2_)_2_[Mo_2_(C_10_H_12_N_2_O_8_)O_6_]·4H_2_O	*F*(000) = 1576
*M**_r_* = 772.40	*D*_x_ = 1.853 Mg m^−^^3^
Monoclinic, *C*2/*c*	Mo *K*α radiation, λ = 0.71073 Å
*a* = 22.5897 (14) Å	Cell parameters from 9873 reflections
*b* = 7.5100 (4) Å	θ = 2.9–27.5°
*c* = 16.3743 (10) Å	µ = 1.00 mm^−^^1^
β = 94.716 (2)°	*T* = 150 K
*V* = 2768.5 (3) Å^3^	Block, colourless
*Z* = 4	0.17 × 0.17 × 0.13 mm

### Bis(ethylenediammonium) µ-ethylenediaminetetraacetato-1κ3O,N,O':2κ3O'',N',O'''-bis[trioxidomolybdate(VI)] tetrahydrate Data collection

**Table:**

Bruker APEXII CCD diffractometer	2946 reflections with *I* > 2σ(*I*)
φ and ω scans	*R*_int_ = 0.032
Absorption correction: multi-scan (SADABS; Krause *et al.*, 2015)	θ_max_ = 27.5°, θ_min_ = 1.8°
*T*_min_ = 0.691, *T*_max_ = 0.746	*h* = −29→29
24031 measured reflections	*k* = −9→9
3192 independent reflections	*l* = −20→21

### Bis(ethylenediammonium) µ-ethylenediaminetetraacetato-1κ3O,N,O':2κ3O'',N',O'''-bis[trioxidomolybdate(VI)] tetrahydrate Refinement

**Table:**

Refinement on *F*^2^	Primary atom site location: dual
Least-squares matrix: full	Secondary atom site location: difference Fourier map
*R*[*F*^2^ > 2σ(*F*^2^)] = 0.022	Hydrogen site location: inferred from neighbouring sites
*wR*(*F*^2^) = 0.060	H-atom parameters constrained
*S* = 1.06	*w* = 1/[σ^2^(*F*_o_^2^) + (0.0247*P*)^2^ + 9.3382*P*] where *P* = (*F*_o_^2^ + 2*F*_c_^2^)/3
3192 reflections	(Δ/σ)_max_ = 0.001
189 parameters	Δρ_max_ = 0.67 e Å^−^^3^
0 restraints	Δρ_min_ = −1.06 e Å^−^^3^

### Bis(ethylenediammonium) µ-ethylenediaminetetraacetato-1κ3O,N,O':2κ3O'',N',O'''-bis[trioxidomolybdate(VI)] tetrahydrate Fractional atomic coordinates and isotropic or equivalent isotropic displacement parameters (Å^2^)

**Table:**

	*x*	*y*	*z*	*U*_iso_*/*U*_eq_	
Mo1	0.34131 (2)	0.30046 (2)	0.43898 (2)	0.01140 (6)	
O2	0.38304 (7)	0.07064 (19)	0.38526 (10)	0.0184 (3)	
O3	0.34930 (6)	0.3750 (2)	0.31149 (9)	0.0153 (3)	
O5	0.32695 (7)	0.5278 (2)	0.45742 (9)	0.0167 (3)	
O6	0.27328 (7)	0.1984 (2)	0.41260 (10)	0.0192 (3)	
O7	0.36649 (7)	0.2195 (2)	0.53376 (10)	0.0199 (3)	
O8	0.46292 (8)	−0.0988 (2)	0.38094 (12)	0.0305 (4)	
O9	0.40393 (8)	0.4592 (3)	0.21238 (10)	0.0313 (4)	
N1	0.44444 (7)	0.3699 (2)	0.42662 (10)	0.0110 (3)	
N2	0.68095 (9)	−0.1480 (2)	0.31790 (11)	0.0180 (4)	
H2C	0.682765	−0.194661	0.266874	0.022*	
H2D	0.644495	−0.169814	0.335592	0.022*	
H2E	0.709362	−0.199255	0.352923	0.022*	
N3	0.68986 (8)	0.3285 (2)	0.39152 (11)	0.0166 (4)	
H3C	0.662370	0.374203	0.353458	0.020*	
H3D	0.726961	0.352136	0.376498	0.020*	
H3E	0.685339	0.379149	0.441115	0.020*	
C1	0.43883 (10)	0.0440 (3)	0.39471 (13)	0.0165 (4)	
C2	0.46667 (9)	0.4795 (3)	0.49874 (12)	0.0136 (4)	
H2A	0.458550	0.415662	0.549512	0.016*	
H2B	0.444417	0.593202	0.497625	0.016*	
C3	0.44903 (9)	0.4682 (3)	0.34868 (12)	0.0158 (4)	
H3A	0.486764	0.435686	0.325738	0.019*	
H3B	0.450105	0.597629	0.360192	0.019*	
C4	0.68156 (10)	0.1326 (3)	0.39721 (13)	0.0181 (4)	
H4A	0.640934	0.106237	0.412324	0.022*	
H4B	0.710243	0.083077	0.440266	0.022*	
C5	0.39759 (10)	0.4287 (3)	0.28535 (13)	0.0159 (4)	
C6	0.47677 (9)	0.1985 (3)	0.42775 (15)	0.0184 (4)	
H6A	0.492092	0.171560	0.484797	0.022*	
H6B	0.511335	0.210675	0.394705	0.022*	
C7	0.69112 (12)	0.0470 (3)	0.31517 (13)	0.0225 (5)	
H7A	0.663501	0.100557	0.271922	0.027*	
H7B	0.732207	0.070407	0.301180	0.027*	
O1	0.30869 (7)	−0.2039 (2)	0.34909 (9)	0.0180 (3)	
H1A	0.317459	−0.290614	0.383210	0.027*	
H1B	0.333866	−0.120160	0.364109	0.027*	
O10	0.56206 (14)	−0.0882 (7)	0.30040 (19)	0.1003 (14)	
H10A	0.533675	−0.115649	0.330976	0.151*	
H10B	0.549760	−0.103261	0.249098	0.151*	

### Bis(ethylenediammonium) µ-ethylenediaminetetraacetato-1κ3O,N,O':2κ3O'',N',O'''-bis[trioxidomolybdate(VI)] tetrahydrate Atomic displacement parameters (Å^2^)

**Table:**

	*U*^11^	*U*^22^	*U*^33^	*U*^12^	*U*^13^	*U*^23^
Mo1	0.01159 (9)	0.01140 (9)	0.01128 (9)	−0.00124 (6)	0.00129 (6)	−0.00028 (6)
O2	0.0146 (7)	0.0131 (7)	0.0272 (8)	−0.0018 (6)	−0.0006 (6)	−0.0051 (6)
O3	0.0139 (7)	0.0207 (7)	0.0113 (7)	−0.0022 (6)	−0.0005 (5)	0.0000 (6)
O5	0.0197 (8)	0.0149 (7)	0.0157 (7)	0.0013 (6)	0.0031 (6)	−0.0022 (6)
O6	0.0142 (7)	0.0206 (8)	0.0229 (8)	−0.0029 (6)	0.0024 (6)	−0.0023 (6)
O7	0.0201 (8)	0.0226 (8)	0.0172 (7)	−0.0033 (6)	0.0021 (6)	0.0059 (6)
O8	0.0238 (9)	0.0153 (8)	0.0507 (12)	0.0053 (7)	−0.0067 (8)	−0.0107 (8)
O9	0.0263 (9)	0.0548 (12)	0.0123 (7)	−0.0165 (8)	−0.0016 (7)	0.0047 (8)
N1	0.0129 (8)	0.0100 (7)	0.0097 (7)	−0.0009 (6)	−0.0016 (6)	−0.0013 (6)
N2	0.0225 (9)	0.0163 (9)	0.0152 (8)	0.0008 (7)	0.0019 (7)	0.0001 (7)
N3	0.0183 (9)	0.0176 (9)	0.0137 (8)	0.0017 (7)	−0.0007 (7)	−0.0025 (7)
C1	0.0174 (10)	0.0132 (9)	0.0185 (10)	0.0003 (8)	−0.0018 (8)	−0.0013 (8)
C2	0.0133 (10)	0.0156 (9)	0.0115 (9)	−0.0025 (7)	−0.0017 (7)	−0.0027 (7)
C3	0.0150 (10)	0.0198 (10)	0.0125 (9)	−0.0062 (8)	0.0001 (7)	0.0020 (8)
C4	0.0234 (11)	0.0170 (10)	0.0138 (9)	0.0003 (8)	0.0019 (8)	−0.0002 (8)
C5	0.0190 (10)	0.0160 (10)	0.0125 (9)	−0.0031 (8)	−0.0002 (8)	0.0000 (8)
C6	0.0130 (10)	0.0141 (10)	0.0273 (11)	0.0015 (8)	−0.0038 (8)	−0.0038 (8)
C7	0.0376 (13)	0.0159 (10)	0.0138 (10)	−0.0012 (9)	0.0013 (9)	−0.0001 (8)
O1	0.0207 (8)	0.0152 (7)	0.0175 (7)	−0.0052 (6)	−0.0025 (6)	0.0023 (6)
O10	0.0533 (18)	0.200 (4)	0.0469 (16)	0.015 (2)	0.0000 (14)	0.007 (2)

### Bis(ethylenediammonium) µ-ethylenediaminetetraacetato-1κ3O,N,O':2κ3O'',N',O'''-bis[trioxidomolybdate(VI)] tetrahydrate Geometric parameters (Å, º)

**Table:**

Mo1—O2	2.1858 (15)	N3—C4	1.487 (3)
Mo1—O3	2.1831 (14)	C1—C6	1.516 (3)
Mo1—O5	1.7686 (15)	C2—C2^i^	1.534 (4)
Mo1—O6	1.7397 (15)	C2—H2A	0.9900
Mo1—O7	1.7195 (16)	C2—H2B	0.9900
Mo1—N1	2.4121 (17)	C3—H3A	0.9900
O2—C1	1.273 (3)	C3—H3B	0.9900
O3—C5	1.270 (3)	C3—C5	1.521 (3)
O8—C1	1.232 (3)	C4—H4A	0.9900
O9—C5	1.236 (3)	C4—H4B	0.9900
N1—C2	1.492 (2)	C4—C7	1.521 (3)
N1—C3	1.485 (3)	C6—H6A	0.9900
N1—C6	1.479 (3)	C6—H6B	0.9900
N2—H2C	0.9100	C7—H7A	0.9900
N2—H2D	0.9100	C7—H7B	0.9900
N2—H2E	0.9100	O1—H1A	0.8703
N2—C7	1.484 (3)	O1—H1B	0.8697
N3—H3C	0.9100	O10—H10A	0.8701
N3—H3D	0.9100	O10—H10B	0.8702
N3—H3E	0.9100		
			
O2—Mo1—N1	71.68 (6)	O8—C1—C6	119.11 (19)
O3—Mo1—O2	75.25 (6)	N1—C2—C2^i^	113.4 (2)
O3—Mo1—N1	73.02 (5)	N1—C2—H2A	108.9
O5—Mo1—O2	157.26 (6)	N1—C2—H2B	108.9
O5—Mo1—O3	86.93 (6)	C2^i^—C2—H2A	108.9
O5—Mo1—N1	89.85 (6)	C2^i^—C2—H2B	108.9
O6—Mo1—O2	87.34 (6)	H2A—C2—H2B	107.7
O6—Mo1—O3	90.94 (7)	N1—C3—H3A	109.1
O6—Mo1—O5	107.27 (7)	N1—C3—H3B	109.1
O6—Mo1—N1	156.07 (6)	N1—C3—C5	112.68 (16)
O7—Mo1—O2	87.85 (7)	H3A—C3—H3B	107.8
O7—Mo1—O3	155.03 (7)	C5—C3—H3A	109.1
O7—Mo1—O5	103.83 (7)	C5—C3—H3B	109.1
O7—Mo1—O6	106.75 (7)	N3—C4—H4A	109.8
O7—Mo1—N1	84.38 (7)	N3—C4—H4B	109.8
C1—O2—Mo1	122.03 (13)	N3—C4—C7	109.60 (17)
C5—O3—Mo1	123.00 (13)	H4A—C4—H4B	108.2
C2—N1—Mo1	108.51 (11)	C7—C4—H4A	109.8
C3—N1—Mo1	108.40 (12)	C7—C4—H4B	109.8
C3—N1—C2	111.30 (15)	O3—C5—C3	117.47 (18)
C6—N1—Mo1	106.85 (12)	O9—C5—O3	123.7 (2)
C6—N1—C2	109.66 (16)	O9—C5—C3	118.68 (19)
C6—N1—C3	111.95 (16)	N1—C6—C1	113.43 (17)
H2C—N2—H2D	109.5	N1—C6—H6A	108.9
H2C—N2—H2E	109.5	N1—C6—H6B	108.9
H2D—N2—H2E	109.5	C1—C6—H6A	108.9
C7—N2—H2C	109.5	C1—C6—H6B	108.9
C7—N2—H2D	109.5	H6A—C6—H6B	107.7
C7—N2—H2E	109.5	N2—C7—C4	110.94 (18)
H3C—N3—H3D	109.5	N2—C7—H7A	109.5
H3C—N3—H3E	109.5	N2—C7—H7B	109.5
H3D—N3—H3E	109.5	C4—C7—H7A	109.5
C4—N3—H3C	109.5	C4—C7—H7B	109.5
C4—N3—H3D	109.5	H7A—C7—H7B	108.0
C4—N3—H3E	109.5	H1A—O1—H1B	104.5
O2—C1—C6	116.65 (18)	H10A—O10—H10B	109.4
O8—C1—O2	124.2 (2)		
			
Mo1—O2—C1—O8	164.21 (18)	N1—C3—C5—O3	24.0 (3)
Mo1—O2—C1—C6	−13.7 (3)	N1—C3—C5—O9	−159.7 (2)
Mo1—O3—C5—O9	174.61 (18)	N3—C4—C7—N2	−177.90 (18)
Mo1—O3—C5—C3	−9.3 (3)	C2—N1—C3—C5	−143.96 (17)
Mo1—N1—C2—C2^i^	175.59 (18)	C2—N1—C6—C1	145.79 (18)
Mo1—N1—C3—C5	−24.7 (2)	C3—N1—C2—C2^i^	−65.2 (3)
Mo1—N1—C6—C1	28.4 (2)	C3—N1—C6—C1	−90.2 (2)
O2—C1—C6—N1	−13.0 (3)	C6—N1—C2—C2^i^	59.2 (3)
O8—C1—C6—N1	169.0 (2)	C6—N1—C3—C5	92.9 (2)

Symmetry code: (i) −*x*+1, −*y*+1, −*z*+1.

### Bis(ethylenediammonium) µ-ethylenediaminetetraacetato-1κ3O,N,O':2κ3O'',N',O'''-bis[trioxidomolybdate(VI)] tetrahydrate Hydrogen-bond geometry (Å, º)

**Table:**

*D*—H···*A*	*D*—H	H···*A*	*D*···*A*	*D*—H···*A*
N2—H2*C*···O1^ii^	0.91	1.93	2.795 (2)	159
N2—H2*D*···O10	0.91	2.00	2.715 (4)	134
N2—H2*D*···O7^iii^	0.91	2.21	2.786 (2)	121
N2—H2*E*···O6^iv^	0.91	1.84	2.748 (2)	172
N3—H3*C*···O9^ii^	0.91	1.88	2.785 (3)	170
N3—H3*D*···O1^v^	0.91	1.98	2.838 (2)	156
N3—H3*E*···O5^i^	0.91	1.84	2.753 (2)	177
O1—H1*A*···O5^vi^	0.87	1.83	2.694 (2)	173
O1—H1*B*···O2	0.87	1.83	2.694 (2)	173
O10—H10*A*···O8	0.87	1.86	2.692 (4)	160
O10—H10*B*···O8^ii^	0.87	2.13	2.978 (4)	166

Symmetry codes: (i) −*x*+1, −*y*+1, −*z*+1; (ii) −*x*+1, *y*, −*z*+1/2; (iii) −*x*+1, −*y*, −*z*+1; (iv) *x*+1/2, *y*−1/2, *z*; (v) *x*+1/2, *y*+1/2, *z*; (vi) *x*, *y*−1, *z*.
